# An analysis of the associations between gender and metabolic syndrome components in Korean adults: a national cross-sectional study

**DOI:** 10.1186/s12902-019-0393-0

**Published:** 2019-06-27

**Authors:** Young-Mo Yang, Byung-Cheul Shin, Chihyoung Son, In-Hyuk Ha

**Affiliations:** 1 Department of Biomedical Informatics, Ajou University School of Medicine, 164, Worldcup-Ro, Yeongtong-gu, Suwon, 16499 Korea; 2 Department of Korean Rehabilitation Medicine, Pusan National University Korean Medicine Hospital, Yangsan, Republic of Korea; 3 Department of Oriental Rehabilitation Medicine, National Rehabilitation Center, Seoul, Republic of Korea; 4 Jaseng Spine and Joint Research Institute, Jaseng Medical Foundation, 3F JS Tower, 538 Gangnam-daero, Gangnam-gu, Seoul, 06110 Republic of Korea

**Keywords:** Metabolic syndrome, Koreans, Gender, Cross-sectional study, Menopause

## Abstract

**Background:**

This study aimed to examine the associations between gender and the prevalence of metabolic syndrome (MS) components among Korean adults by age and body mass index (BMI) subgroups.

**Methods:**

This study obtained data from the sixth Korea National Health and Nutrition Examination Survey 2013–2015, a cross-sectional and nationally representative survey conducted by the Korean Centers for Diseases Control and Prevention.

**Results:**

Of the 11,136 subjects included in this study, there were 4627 (41.5%) men and 6509 (58.5%) women. Compared to women, men were at higher risks of hypertension (HTN) (odds ratio [OR], 1.508; 95% confidence interval [CI], 1.320–1.723), diabetes mellitus (DM) (OR, 1.638; 95% CI, 1.333–2.013), prediabetes (OR, 1.549; 95% CI, 1.355–1.771), and hypertriglyceridemia (OR, 2.466; 95% CI, 2.097–2.900), but at lower risks of low high-density lipoprotein (HDL) (OR, 0.346; 95% CI, 0.307–0.390) and high waist circumference (WC) (OR, 0.780; 95% CI, 0.647–0.940). Among subjects with BMI < 25 kg/m^2^, the risks of HTN, DM, prediabetes, and hypertriglyceridemia were higher in men than in women, whereas the risks of low HDL level and high WC were lower in men. Similarly, among subjects with BMI ≥25 kg/m^2^, compared to women, men were at higher risks of HTN, DM, prediabetes, and hypertriglyceridemia, but at lower risks of low HDL level.

**Conclusions:**

The difference in the prevalence of MS components between men and women can be partially explained by the different effects of gender on the etiology of MS components. The results showed that gender was likely to contribute to an increase in the prevalence of MS components. HTN, DM, prediabetes, and hypertriglyceridemia were more prevalent in men than in women, whereas the prevalence of low HDL level and high WC were higher in women than in men. Similar results were found in subgroup analyses by age and BMI.

## Background

Metabolic syndrome (MS) is a cluster of metabolic abnormalities. The MS-associated factors including waist circumference (WC), triglyceride (TG) levels, high-density lipoprotein (HDL) cholesterol levels, hypertension (HTN), and fasting blood glucose (FBG) levels are classic risk factors for cardiovascular disease and diabetes mellitus (DM) [1, 2]. The prevalence rates of MS varied by geographical regions; however, it was estimated that approximately 25% of adults had MS in most countries [3–5].

Over the past decades, the prevalence of MS rapidly increased in Asian countries including Korea [5–8]. Li and colleagues reported that the overall prevalence rate of MS in China was 24.2% (24.6% in men and 23.8% in women) [5]. In Eastern India, the overall prevalence rate of MS was 33.5% (24.9% in men and 42.3% in women) [8]. In Korea, MS was reported in 26.9% of the entire population (30.0% in men and 24.6% in women) [9].

Additionally, there was a gender-related disparity in the prevalence of MS. It tended to occur more frequently in men than in women. However, a reversed trend was shown among old adults, as reported in several studies [1, 5, 10–13]. This tendency may be explained by the gender differences in the prevalence of MS-related risk factors. According to previous studies, high blood pressure (BP), high level of TG, and elevated level of FBG were more prevalent in men, whereas low HDL level and high WC were more prevalent in women [4, 10].

Consequently, gender as an independent risk factor for the components of MS may play an important role in determining the prevalence of MS in men and women. However, limited studies investigated the relationships between gender and the prevalence of MS components among Korean adults [10]. Therefore, this study aims to examine the associations between gender and the prevalence of MS components in Korean adults by age and body mass index (BMI) subgroups.

## Methods

### Study population

**Fig. 1:**
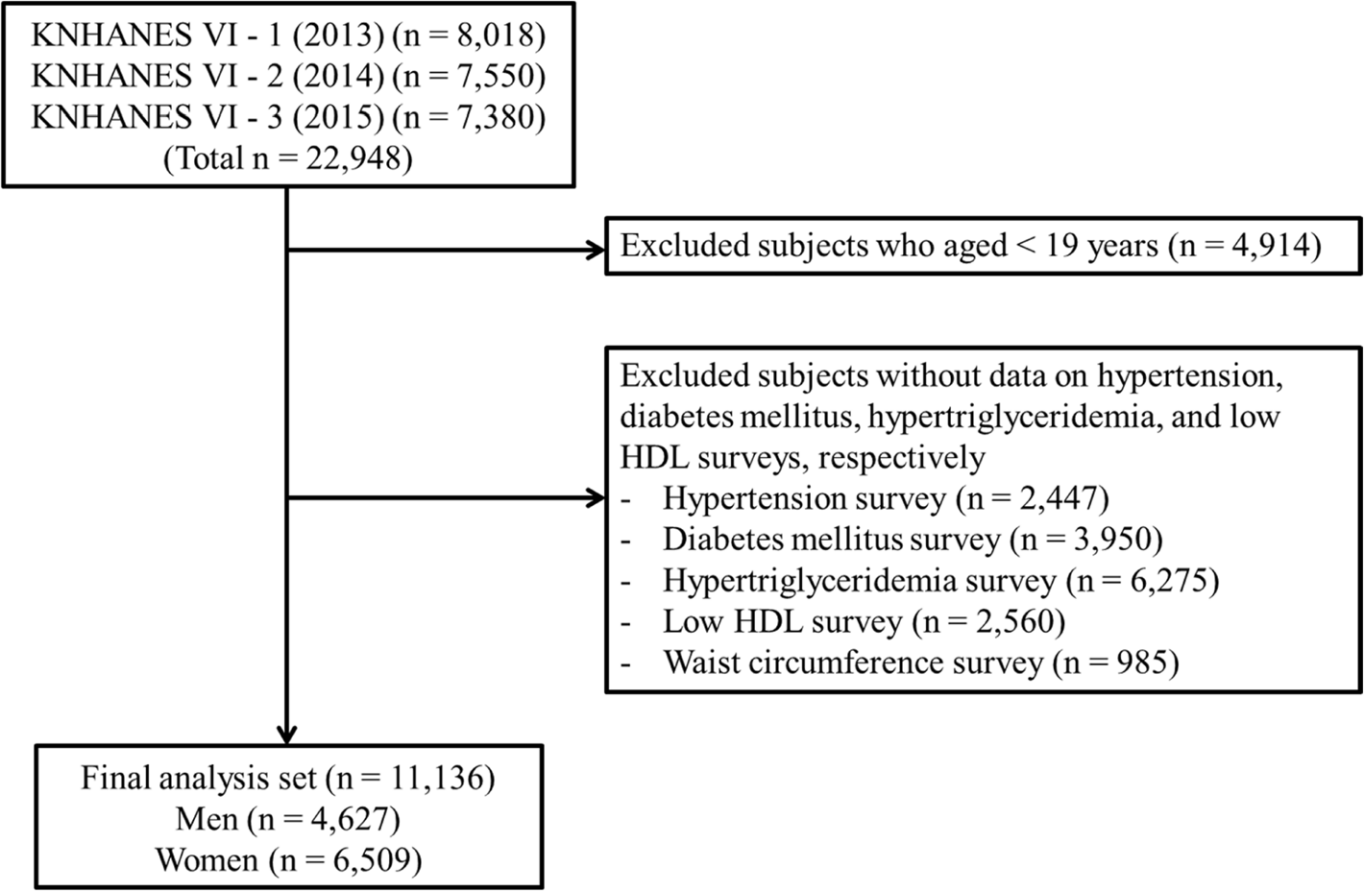
Subject selection from the Korea National Health and Nutrition Examination Survey 2013–2015. KNHANES, Korea national health and nutrition examination survey; HDL, high-density lipoprotein

This study obtained data from the sixth Korea National Health and Nutrition Examination Survey (KNHANES VI) (2013–2015), a cross-sectional and nationally representative survey conducted by the Korean Centers for Diseases Control and Prevention (CDC). The KNHANES data were collected every year from 3840 individuals randomly selected from 192 regions in Korea using a stratified multi-stage probability sampling design. The KNHANES consisted of a health interview, a health examination, and a nutrition survey. The data were obtained through household interviews, and the standardized physical examinations were carried out at mobile examination centers. Written informed consent was obtained from all study participants. This study was approved by the Korean CDC Institutional Review Board. All study subjects were aged ≥19 years. Only subjects without missing data on hypertension, diabetes mellitus, hypertriglyceridemia, and low HDL level were eligible for this study. Ultimately, 11,136 adults (4627 men and 6509 women) were included in this study (Fig. 1).

### Blood tests

Fasting serum glucose, triglyceride, and HDL cholesterol levels were measured after an overnight (at least 8–12 h) fasting period. Subjects were categorized into three groups based on the measured fasting glucose levels: normal glucose level (< 100 mg/dL), impaired fasting glucose or prediabetes (100–125 mg/dL), and DM (≥126 mg/dL) [14]. All subjects with a known diagnosis of DM treated with antiglycemic agents and/or insulin were assigned to the DM group, regardless of their fasting glucose levels. Hypertriglyceridemia was defined as fasting triglyceride level ≥ 200 mg/dL, which corresponds to a high or very high TG level according to the National Cholesterol Education Program Adult Treatment Panel III (NCEP ATP III) guidelines [15]. Low HDL cholesterol level was defined as HDL level < 40 mg/dL for men and HDL level < 50 mg/dL for women [15].

### Blood pressure measurements

Study subjects were seated for at least 5 min before the measurement of blood pressure on the right arm with a standard mercury sphygmomanometer. Three measurements were taken for each subject at 5-min intervals. The average of the second and third measurements was used in the analysis. Hypertension was defined as systolic blood pressure (SBP) ≥140 mmHg, or diastolic blood pressure (DBP) ≥90 mmHg, or if subjects were under antihypertensive therapy [16].

### Body mass index and waist circumference

Body weight and height were measured to the nearest 0.1 kg and 0.1 cm. Study subjects were required to wear light indoor clothing without shoes. BMI was computed by dividing weight in kilograms by height in meters squared (kg/m^2^). Subjects were categorized into three groups by the BMI: underweight (BMI < 18.5 kg/m^2^), normal weight (BMI: 18.5–25.0 kg/m^2^), and overweight or obese (BMI ≥25.0 kg/m^2^) [17]. Waist circumference (WC) measurement was taken to the nearest 0.1 cm in a horizontal plane at the midpoint between the iliac crest and the lower rib. High WC was defined as WC ≥90 cm for men and WC ≥ 85 cm for women according to the criteria for abdominal obesity recommended by the Korean Society for the Study of Obesity [18].

### Other variables

Self-reported age, socioeconomic characteristics (i.e., household income and educational level), and lifestyle risk factors (i.e., smoking status, alcohol consumption, and sedentary time) were collected from the questionnaire survey. The average monthly household income was categorized by quartiles into low, lower middle, higher middle, and high groups. Educational level was categorized into four groups: elementary school graduation or lower, middle school graduation, high school graduation, and college graduation or higher. By their self-reported smoking status, subjects were categorized in to current smokers (who had smoked ≥100 cigarettes and kept smoking at the time of the survey), or non−/ex-smokers (who had never smoked or had smoked < 100 cigarettes in their lifetime/those who had smoked ≥100 cigarettes, but did not smoke at the time of the survey). Alcohol consumption was dichotomized into zero consumption and non-zero consumption. Sedentary time was dichotomized using a cut-off of 7 h/day [19].

### Statistical analysis

SAS version 9.4 (SAS Institute Inc., Cary, NC, USA) survey procedure was utilized for all statistical analyses. The KNHANES sampling weights were applied to obtain nationally representative estimates. Data analysis was conducted by applying a complex sampling design with stratified variables, cluster variables, and weighted variables. *P*-value of 0.05 was used to define statistical significance. Subgroup analyses were performed by gender and age subgroups (19–54 years and ≥ 55 years). Categorical variables were presented as frequency and percentage (%), whereas continuous variables were reported as mean and standard deviation. Chi-square tests and independent t-test were applied wherever appropriate. Multivariate logistic regression analysis was used to evaluate the impact of gender (using women as the reference group) on the prevalence of MS components by age and BMI subgroups. The results were reported as odds ratios (ORs) and 95% confidence intervals (CIs).

## Results

Of the 11,136 subjects included in this study, there were 4627 men (41.5%) and 6509 women (58.5%). The characteristics of these participants are summarized in Table 1. Compared to women, the prevalence of HTN, DM, prediabetes, hypertriglyceridemia, and high WC were higher in men. However, the prevalence of low HDL level was lower in men than in women. All the differences were statistically significant (*p* <  0.0001 for all). Similar results were shown in subgroup analyses by age groups. Furthermore, aside from the MS components, there were statistically significant differences in other variables between men and women.

**Table 1:** Characteristics of the study population

Characteristic	Total			19–54 years			≥ 55 years		
	Men (*n* = 4627)	Women (*n* = 6509)	*p*-value	Men (*n* = 2368)	Women (*n* = 3556)	*p*-value	Men (*n* = 2259)	Women (*n* = 2953)	*p*-value
Age (years), mean (SD)	45.5 ± 16.1	47.0 ± 16.1	< 0.0001	36.4 ± 10.3	37.3 ± 9.9	0.0017	64.2 ± 7.6	64.8 ± 7.9	0.0178
Household income, *n* (%)									
Low	821 (17.8)	1292 (19.9)	< 0.0001	179 (7.6)	257 (7.3)	0.1290	642 (28.6)	1035 (35.2)	< 0.0001
Lower middle	1163 (25.3)	1695 (26.2)		530 (22.5)	889 (25.1)		633 (28.2)	806 (27.4)	
Higher middle	1296 (28.1)	1742 (26.9)		802 (34.0)	1152 (32.5)		494 (22.0)	590 (20.1)	
High	1325 (28.8)	1750 (27.0)		848 (36.0)	1244 (35.1)		477 (21.2)	506 (17.2)	
Educational level, *n* (%)									
≤ Elementary school	801 (17.5)	1781 (27.6)	< 0.0001	61 (2.6)	120 (3.4)	0.0362	740 (33.2)	1661 (56.9)	< 0.0001
Middle school	519 (11.3)	675 (10.5)		93 (4.0)	204 (5.8)		426 (19.1)	471 (16.1)	
High school	1665 (36.3)	2119 (32.8)		1035 (44.0)	1561 (44.1)		630 (28.3)	558 (19.1)	
≥ College	1599 (34.9)	1883 (29.2)		1166 (49.5)	1654 (46.7)		433 (19.4)	229 (7.9)	
Height (m), mean (SD)	170.9 ± 6.7	157.7 ± 6.4	< 0.0001	172.9 ± 6.1	159.9 ± 5.7	< 0.0001	166.8 ± 5.9	153.7 ± 5.8	< 0.0001
Weight (kg), mean (SD)	71.2 ± 11.7	57.8 ± 9.2	< 0.0001	73.3 ± 12.0	57.9 ± 9.6	< 0.0001	66.8 ± 9.7	57.5 ± 8.5	< 0.0001
BMI (kg/m^2^), mean (SD)	24.3 ± 3.4	23.2 ± 3.6	< 0.0001	24.5 ± 3.6	22.7 ± 3.6	< 0.0001	24.0 ± 2.9	24.3 ± 3.2	0.0007
< 18.5 (underweight), *n* (%)	117 (2.5)	316 (4.9)	< 0.0001	52 (2.2)	265 (7.5)	< 0.0001	65 (2.9)	51 (1.7)	0.0324
< 25 (normoweight), *n* (%)	2772 (60.0)	4267 (65.9)		1350 (57.1)	2508 (71.2)		1422 (63.0)	1759 (59.6)	
≥ 25 (overweight or obese), *n* (%)	1733 (37.5)	1893 (29.2)		963 (40.7)	752 (21.3)		770 (34.1)	1141 (38.7)	
Smokers, *n* (%)									
Non−/Ex-smoker	2954 (64.6)	6163 (95.7)	< 0.0001	1344 (57.0)	3349 (94.3)	< 0.0001	1610 (72.7)	2814 (97.4)	< 0.0001
Current smoker	1620 (35.4)	277 (4.3)		1016 (43.1)	202 (5.7)		604 (27.3)	75 (2.6)	
Alcohol consumption, *n* (%)									
No	237 (5.2)	1165 (18.1)	< 0.0001	75 (3.2)	291 (8.2)	< 0.0001	162 (7.3)	874 (30.2)	< 0.0001
Yes	4342 (94.8)	5285 (81.9)		2285 (96.8)	3260 (91.8)		2057 (92.7)	2025 (69.9)	
Waist circumference (cm), mean (SD)	84.8 ± 9.2	77.9 ± 9.7	< 0.0001	84.4 ± 9.4	75.6 ± 9.3	< 0.0001	85.6 ± 8.5	82.0 ± 9.1	< 0.0001
High waist circumference, *n* (%)	1289 (27.9)	1605 (24.7)	< 0.0001	621 (26.2)	531 (14.9)	< 0.0001	668 (29.6)	1074 (36.4)	0.0003
Systolic BP (mmHg), mean (SD)	119.3 ± 14.6	113.9 ± 16.7	< 0.0001	116.6 ± 12.7	107.9 ± 13.0	< 0.0001	124.7 ± 16.7	124.7 ± 17.3	0.9926
Diastolic BP (mmHg), mean (SD)	77.7 ± 10.4	72.6 ± 9.7	< 0.0001	78.3 ± 10.3	71.6 ± 9.4	< 0.0001	76.4 ± 10.5	74.4 ± 10.1	< 0.0001
HTN, *n* (%)	1618 (35.0)	1798 (27.6)	< 0.0001	471 (19.9)	306 (8.6)	< 0.0001	1147 (50.8)	1492 (50.5)	0.9814
Fasting glucose level (mg/dL), mean (SD)	100.3 ± 22.2	96.4 ± 20.3	< 0.0001	97.2 ± 20.4	92.7 ± 16.8	< 0.0001	106.7 ± 24.2	103.1 ± 24.1	< 0.0001
Glucose metabolism status, *n* (%)									
Normal	2733 (59.1)	4671 (71.8)	< 0.0001	1715 (72.4)	2987 (84.0)	< 0.0001	1018 (45.1)	1684 (57.0)	< 0.0001
Prediabetes	1256 (27.2)	1192 (18.3)		510 (21.5)	450 (12.7)		746 (33.0)	742 (25.1)	
DM	638 (13.8)	646 (9.9)		143 (6.0)	119 (3.4)		495 (21.9)	527 (17.9)	
Total cholesterol (mg/dL), mean (SD)	188.4 ± 35.9	189.7 ± 35.0	0.1211	190.0 ± 36.1	184.8 ± 32.7	< 0.0001	185.1 ± 35.3	198.6 ± 37.2	< 0.0001
Triglycerides (mg/dL), mean (SD)	156.2 ± 120.5	110.4 ± 74.0	< 0.0001	157.9 ± 126.3	98.5 ± 70.2	< 0.0001	152.8 ± 107.6	132.2 ± 75.7	< 0.0001
LDL cholesterol (mg/dL), mean (SD)	115.7 ± 32.7	115.5 ± 31.8	0.8742	118.5 ± 32.6	112.0 ± 29.8	< 0.0001	110.1 ± 32.4	120.9 ± 34.0	< 0.0001
HDL cholesterol (mg/dL), mean (SD)	47.7 ± 11.1	54.6 ± 12.3	< 0.0001	48.2 ± 10.9	56.3 ± 12.2	< 0.0001	46.7 ± 11.6	51.3 ± 12.0	< 0.0001
Hypertriglyceridemia, *n* (%)	979 (21.2)	668 (10.3)	< 0.0001	551 (23.3)	263 (7.4)	< 0.0001	428 (19.0)	405 (13.7)	< 0.0001
Low HDL cholesterol, *n* (%)	1175 (25.4)	2708 (41.6)	< 0.0001	533 (22.5)	1176 (33.1)	< 0.0001	642 (28.4)	1532 (51.9)	< 0.0001
Sedentary time (hour/day), mean (SD)	7.3 ± 3.7	6.9 ± 3.6	< 0.0001	7.5 ± 3.7	7.2 ± 3.6	0.0028	6.7 ± 3.7	6.4 ± 3.4	0.0266
≤ 7, *n* (%)	2545 (55.9)	3801 (59.6)	0.0002	1171 (49.8)	1979 (55.8)	0.0011	1374 (62.4)	1822 (64.4)	0.0998
> 7, *n* (%)	2008 (44.1)	2574 (40.4)		1179 (50.2)	1565 (44.2)		829 (37.6)	1008 (35.6)	

Abbreviations: *SD* standard deviation, *BMI* body mass index, *BP* blood pressure, *HTN* hypertension, *DM* diabetes mellitus, *LDL* low-density lipoprotein, *HDL* high-density lipoprotein

To determine the associations between gender and the prevalence of MS components in all subjects, logistic regression analysis was performed. The results are presented in Table 2. Compared to women, men were at higher risks of HTN, DM, prediabetes, and hypertriglyceridemia, but lower risks of low HDL level and high WC. In subjects with BMI < 25 kg/m^2^, the risks of developing HTN, DM, prediabetes, and hypertriglyceridemia were higher in men than in women, whereas the risks of low HDL level and high WC were lower in men. Similarly, in subjects with BMI ≥25 kg/m^2^, the risks of HTN, DM, prediabetes, and hypertriglyceridemia were higher in men than in women, whereas the risk of low HDL level was lower in men.

**Table 2:** Odds ratios for the prevalence of metabolic syndrome components among study subjects

Metabolic syndrome components	Unadjusted OR (95% CI)	*p*-value	Adjusted OR (95% CI)	*p*-value	Adjusted OR (95% CI) for BMI < 25 kg/m^2a^	*p*-value	Adjusted OR (95% CI) for BMI ≥ 25 kg/m^2a^	*p*-value
HTN^b^	1.373 (1.256–1.501)	< 0.0001	1.508 (1.320–1.723)	< 0.0001	1.331 (1.111–1.595)	0.0020	1.716 (1.383–2.131)	< 0.0001
DM^c^	1.543 (1.333–1.785)	< 0.0001	1.638 (1.333–2.013)	< 0.0001	1.847 (1.425–2.395)	< 0.0001	1.444 (1.060–1.968)	0.0201
Prediabetes^c^	1.708 (1.536–1.899)	< 0.0001	1.549 (1.355–1.771)	< 0.0001	1.760 (1.468–2.111)	< 0.0001	1.337 (1.072–1.668)	0.0102
Hypertriglyceridemia^d^	2.785 (2.466–3.146)	< 0.0001	2.466 (2.097–2.900)	< 0.0001	2.071 (1.639–2.617)	< 0.0001	3.060 (2.449–3.824)	< 0.0001
Low HDL^e^	0.503 (0.458–0.553)	< 0.0001	0.346 (0.307–0.391)	< 0.0001	0.369 (0.313–0.435)	< 0.0001	0.304 (0.251–0.368)	< 0.0001
High waist circumference^f^	1.288 (1.167–1.421)	< 0.0001	0.780 (0.647–0.940)	0.0093	0.705 (0.534–0.930)	0.0136	0.977 (0.800–1.193)	0.8160

Notes: Odds ratios with adjustments using logistic regression models

^a^Not adjusted for BMI

^b^Adjusted for age, household income, educational level, BMI, smoking status, alcohol consumption, DM, prediabetes, hypertriglyceridemia, low HDL level, high waist circumference, and sedentary time

^c^Adjusted for age, household income, educational level, BMI, smoking status, alcohol consumption, HTN, hypertriglyceridemia, low HDL level, high waist circumference, and sedentary time

^d^Adjusted for age, household income, educational level, BMI, smoking status, alcohol consumption, HTN, DM, prediabetes, low HDL level, high waist circumference, and sedentary time

^e^Adjusted for age, household income, educational level, BMI, smoking status, alcohol consumption, HTN, DM, prediabetes, hypertriglyceridemia, high waist circumference, and sedentary time

^f^Adjusted for age, household income, educational level, BMI, smoking status, alcohol consumption, HTN, DM, prediabetes, hypertriglyceridemia, low HDL level, and sedentary time

Abbreviations: *HTN* hypertension, *DM* diabetes mellitus, *HDL* high-density lipoprotein, *BMI* body mass index

The results of the logistic regression analysis among subjects aged 19–54 years are presented in Table 3. Compared to women, men were at higher risks of HTN, prediabetes, and hypertriglyceridemia, but lower risks of low HDL level and high WC. No significant difference was found in DM between men and women. In subjects with BMI < 25 kg/m^2^, the risks of HTN, DM, prediabetes, and hypertriglyceridemia were higher in men than in women, whereas the risks of low HDL level was lower in men. In subjects with BMI ≥25 kg/m^2^, compared to women, men were at higher risks of HTN and hypertriglyceridemia but lower risks of low HDL level.

**Table 3:** Odds ratios for the prevalence of metabolic syndrome components among subjects aged 19–54 years

Metabolic syndrome components	Unadjusted OR (95% CI)	*p*-value	Adjusted OR (95% CI)	*p*-value	Adjusted OR (95% CI) for BMI < 25 kg/m^2a^	*p*-value	Adjusted OR (95% CI) for BMI ≥ 25 kg/m^2a^	*p*-value
HTN^b^	2.577 (2.195–3.026)	< 0.0001	1.987 (1.615–2.445)	< 0.0001	1.690 (1.236–2.312)	0.0011	2.209 (1.593–3.063)	< 0.0001
DM^c^	2.169 (1.633–2.881)	< 0.0001	1.421 (0.974–2.073)	0.0686	1.909 (1.163–3.133)	0.0106	1.067 (0.622–1.831)	0.8129
Prediabetes^c^	1.939 (1.665–2.257)	< 0.0001	1.417 (1.169–1.718)	0.0004	1.785 (1.377–2.315)	< 0.0001	1.094 (0.812–1.475)	0.5545
Hypertriglyceridemia^d^	3.879 (3.286–4.579)	< 0.0001	3.234 (2.582–4.051)	< 0.0001	3.238 (2.317–4.524)	< 0.0001	3.803 (2.755–5.250)	< 0.0001
Low HDL^e^	0.581 (0.510–0.661)	< 0.0001	0.311 (0.261–0.371)	< 0.0001	0.375 (0.298–0.472)	< 0.0001	0.243 (0.183–0.321)	< 0.0001
High waist circumference^f^	1.937 (1.691–2.218)	< 0.0001	0.688 (0.517–0.915)	0.0103	0.673 (0.398–1.137)	0.1383	0.946 (0.728–1.230)	0.6801

Notes: Odds ratios with adjustments using logistic regression models

^a^Not adjusted for BMI

^b^Adjusted for age, household income, educational level, BMI, smoking status, alcohol consumption, DM, prediabetes, hypertriglyceridemia, low HDL level, high waist circumference, and sedentary time

^c^Adjusted for age, household income, educational level, BMI, smoking status, alcohol consumption, HTN, hypertriglyceridemia, low HDL level, high waist circumference, and sedentary time

^d^Adjusted for age, household income, educational level, BMI, smoking status, alcohol consumption, HTN, DM, prediabetes, low HDL level, high waist circumference, and sedentary time

^e^Adjusted for age, household income, educational level, BMI, smoking status, alcohol consumption, HTN, DM, prediabetes, hypertriglyceridemia, high waist circumference, and sedentary time

^f^Adjusted for age, household income, educational level, BMI, smoking status, alcohol consumption, HTN, DM, prediabetes, hypertriglyceridemia, low HDL level, and sedentary time

Abbreviations: *HTN* hypertension, *DM* diabetes mellitus, *HDL* high-density lipoprotein, *BMI* body mass index

Finally, the results of the logistic regression analysis among subjects aged ≥55 years are presented in Table 4. Men were at higher risks of HTN, DM, prediabetes, and hypertriglyceridemia, but lower risks for low HDL level than women. In subjects with BMI < 25 kg/m^2^, the risks of HTN, DM, and prediabetes were higher in men than in women, whereas the risks of low HDL level was lower in men. In subjects with BMI ≥25 kg/m^2^, compared to women, men were at higher risks of DM, prediabetes, and hypertriglyceridemia but lower risks of low HDL level.

**Table 4:** Odds ratios for the prevalence of metabolic syndrome components among subjects aged ≥55 years

Metabolic syndrome components	Unadjusted OR (95% CI)	*p*-value	Adjusted OR (95% CI)	*p*-value	Adjusted OR (95% CI) for BMI < 25 kg/m^2a^	*p*-value	Adjusted OR (95% CI) for BMI ≥ 25 kg/m^2a^	*p*-value
HTN^b^	1.002 (0.881–1.139)	0.9814	1.295 (1.095–1.532)	0.0026	1.380 (1.116–1.707)	0.0030	1.185 (0.895–1.569)	0.2350
DM^c^	1.520 (1.272–1.818)	< 0.0001	1.789 (1.430–2.240)	< 0.0001	1.935 (1.454–2.576)	< 0.0001	1.696 (1.203–2.391)	0.0026
Prediabetes^c^	1.662 (1.437–1.923)	< 0.0001	1.697 (1.421–2.026)	< 0.0001	1.770 (1.397–2.242)	< 0.0001	1.615 (1.194–2.185)	0.0019
Hypertriglyceridemia^d^	1.743 (1.467–2.072)	< 0.0001	1.614 (1.280–2.036)	< 0.0001	1.143 (0.816–1.603)	0.4360	2.353 (1.701–3.255)	< 0.0001
Low HDL^e^	0.402 (0.352–0.460)	< 0.0001	0.383 (0.325–0.451)	< 0.0001	0.368 (0.295–0.458)	< 0.0001	0.382 (0.293–0.499)	< 0.0001
High waist circumference^f^	0.772 (0.670–0.889)	0.0003	0.872 (0.695–1.095)	0.2383	0.816 (0.595–1.119)	0.2059	1.051 (0.772–1.432)	0.7502

Notes: Odds ratios with adjustments using logistic regression models

^a^Not adjusted for BMI

^b^Adjusted for age, household income, educational level, BMI, smoking status, alcohol consumption, DM, prediabetes, hypertriglyceridemia, low HDL level, high waist circumference, and sedentary time

^c^Adjusted for age, household income, educational level, BMI, smoking status, alcohol consumption, HTN, hypertriglyceridemia, low HDL level, high waist circumference, and sedentary time

^d^Adjusted for age, household income, educational level, BMI, smoking status, alcohol consumption, HTN, DM, prediabetes, low HDL level, high waist circumference, and sedentary time

^e^Adjusted for age, household income, educational level, BMI, smoking status, alcohol consumption, HTN, DM, prediabetes, hypertriglyceridemia, high waist circumference, and sedentary time

^f^Adjusted for age, household income, educational level, BMI, smoking status, alcohol consumption, HTN, DM, prediabetes, hypertriglyceridemia, low HDL level, and sedentary time

Abbreviations: *HTN* hypertension, *DM* diabetes mellitus, *HDL* high-density lipoprotein, *BMI* body mass index

## Discussion

In this study, we investigated the associations between gender and the prevalence of MS components among Korean adults using data from the KNHANES VI. The results showed that gender appeared to be an independent predictor of the prevalence of the most MS components. While HTN, DM, prediabetes, and hypertriglyceridemia were more prevalent in men than in women, the prevalence of low HDL level and high WC were higher in women than in men. However, it should be noted that the definitions of low HDL level and high WC differ between men and women. Stricter definitions were set in women than in men, which may partially explain the higher prevalence rates of low HDL level and high WC found in women. Similar results were found in subgroup analyses by age and BMI. The differences in the prevalence of MS components may be related to the sex-related characteristics (e.g., biological traits and functional features) and gender-associated determinants (e.g., psychological and cultural habits) [1]. Importantly, the changes in hormone level during and after menopause may contribute to the gender differences in the prevalence of MS components in older age population [1, 20, 21]. Since the studies on the associations between gender and the prevalence of MS components by age and BMI have been rarely implemented in Korea, it is meaningful in that this study could be utilized as a better knowledge on the development of health strategies for managing MS components according to gender.

The most prominent differences between men and women were the prevalence of hypertriglyceridemia and low HDL level. According to a previous study using the KNHANES data between 1998 and 2010, hypertriglyceridemia and low HDL level were the most prevalent dyslipidemia in Korean adults [22]. In the present study, the overall prevalence of hypertriglyceridemia was 2.466 times higher in men than in women. This trend was more prominent in subjects aged between 19 and 54 years (3.234 times) than those aged ≥55 years (1.614 times). The overall prevalence of low HDL level was 2.890 times higher in women than in men. Similarly, this trend was more prominent in subjects aged between 19 and 54 years (3.215 times) than those aged ≥55 years (2.611 times). The high prevalence of the two conditions in Korean adults may be associated with the high carbohydrate diet in Korea (e.g. rice) [22, 23]. According to a review study investigating the effect of carbohydrate consumption on metabolic parameters in diabetic patients, high intake of carbohydrate was associated with higher serum triglyceride levels and lower HDL levels [24]. Similar results were reported in a cross-sectional study of older women [25]. While abdominal obesity assessed by BMI and WC was negatively associated with HDL levels, it was positively associated with triglyceride levels [22, 26]. This is consistent with our findings, where a higher prevalence of hypertriglyceridemia and low HDL level were observed among overweight/obese subjects with BMI ≥ 25 kg/m^2^ compared to their counterparts, irrespective of gender.

To explore the effect of estrogen on the prevalence of MS components, study subjects were categorized into two groups by age using a cut-off of 55 years, at which a majority of Korean women would have reached menopause. The gender differences in the prevalence of hypertriglyceridemia and low HDL level became smaller among subjects aged ≥55 years compared to their counterparts. However, substantial gender differences in the prevalence of hypertriglyceridemia and low HDL level still existed. Therefore, changes in the hormonal level were not the sole reason for the gender differences in the prevalence of hypertriglyceridemia and low HDL level. This tendency may also be associated with some gender-linked disparities such as different patterns of nutrition intake, lifestyle, or stress and different behaviors between men and women [21].

The overall prevalence of DM was 1.638 times higher in men than in women. While the pattern was observed in both subgroups by BMI, the gender differences were smaller among overweight/obese subjects compared to their counterparts. This is consistent with the findings of a previous study conducted in Uganda [27], in which the authors reported that while no correlation was found between BMI and the risk of type 2 DM among men, the prevalence of DM in women significantly increased with BMI. More specifically, over 70% obese women were diagnosed with DM compared to 25% of women with BMI < 20 kg/m^2^ [27]. In addition, according to the Jackson Heart Study, abdominal obesity is associated with an increased fasting plasma glucose level and a higher prevalence of type 2 DM, and the correlation is stronger in women than in men [28, 29].

Interestingly, gender differences in the prevalence of DM increased in subjects aged ≥55 years compared to their counterparts. Additionally, this tendency was more prominent among subjects with BMI ≥ 25 kg/m^2^. This may be partially attributed to the frequent use of hormone replacement therapy (HRT) among postmenopausal women [30]. After menopause, estrogen deficiency would increase the risk of type 2 DM in women due to the changes in insulin secretion, insulin sensitivity, and glucose effectiveness [28, 31]. Testosterone depletion would increase the risks of hyperglycemia and DM in men via its impact on insulin resistance and visceral adiposity [28, 32]. Sex hormone therapies can reduce, to a certain extent, the risks of hyperglycemia and type 2 DM in men and women [31, 33–35]. Thus, the frequent use of HRT in postmenopausal women is likely to decrease the risks of DM, leading to relatively increased ORs among subjects aged ≥55 years, especially those with BMI ≥ 25 kg/m^2^. However, the positive effects of estrogen and testosterone on glucose homeostasis may be expected within their physiological windows [28].

The overall prevalence of HTN was 1.508 times higher in men than in women. The gender difference was more pronounced in the younger age group. These results were consistent with the German Health Examination Survey study. The German study reported while the prevalence of HTN was 19.5 and 11.6% among men and women, respectively, in the 18–54 years age group, the gender differences were smaller among those aged 55–79 years (60.6% in men vs 61.5% in women) [36]. This can be explained by the changes in the estrogen levels after menopause [37]. Estrogen has a protective effect on the cardiovascular system. Estrogen directly activates the nitric oxide synthase in the endothelial cells and may recover and replace the damaged endothelial cells, which ultimately leads to vasodilation. Indirectly, estrogen also has a positive effect on the serum lipid levels through the receptor-mediated activation of the hepatic genes (e.g., Apo-protein genes) [37]. Moreover, estrogen and ageing may affect salt sensitivity, which is related to blood pressure regulation. After menopause, women become more salt-sensitive, thus more prone to high blood pressure [38].

Additionally, among subjects aged between 19 and 54 years, the disparity in the prevalence of HTN between men and women was more distinct among those with BMI ≥ 25 kg/m^2^. This tendency may be partially explained by the difference in the pattern of fat disposition between men and women. Fat accumulation in women tends to appear more frequently in the lower body areas such as the gluteal site, whereas men show the tendency to accumulate fat in the intra-abdominal sites [39]. This result is likely to contribute to the differences in the prevalence of HTN among this age group because visceral fat is positively related with HTN and insulin resistance [40, 41]. However, this tendency disappeared among subjects aged ≥55 years. This may be associated with estrogen deficiency and the accumulation of fat after menopause in women.

There are some limitations warrant mentioning. First, the cross-sectional design of this study made it difficult to conclude a causal inference between gender and the prevalence of MS components. Second, many variables measured at a single time point were used to assess the effects of gender on the prevalence of MS components, which would have a negative impact on data accuracy. Third, the sociodemographic characteristics of the study population were collected through the survey; thus, this might draw recall bias. Finally, the overall prevalence of MS components was likely to be underestimated because we excluded subjects with incomplete information on MS components. However, this process was unlikely to have a significant impact on the study results because it is highly possible that the missingness occurred at random.

## Conclusions

The differences in the prevalence of MS between men and women can be partially explained by the different effects of gender on MS components. The results showed that gender was likely to contribute to an increased prevalence of MS components. HTN, DM, prediabetes, and hypertriglyceridemia were more prevalent in men than in women, whereas the prevalence of low HDL level and high WC were higher in women than in men. Similar results were observed in subgroup analysis by age and BMI. This trend was attenuated after women reached menopausal age.

## Data Availability

The datasets used during this study are available from the corresponding author on reasonable request.

## Abbreviations

BMI: Body mass index
BP: Blood pressure
CDC: Centers for Diseases Control and Prevention
CI: Confidence interval
DBP: Diastolic blood pressure
DM: Diabetes mellitus
FBG: Fasting blood glucose
HDL: High-density lipoprotein
HRT: Hormone replacement therapy
HTN: Hypertension
KNHANES: Korea National Health and Nutrition Examination Survey
MS: Metabolic syndrome
NCEP ATP III: National Cholesterol Education Program Adult Treatment Panel III
ORs: Odds ratios
SBP: Systolic blood pressure
TG: Triglyceride
WC: Waist circumference
